# Neurosurgery Advancements: From Technical Innovation to Patient-Centered Outcomes—A Narrative Review

**DOI:** 10.3390/jcm15083140

**Published:** 2026-04-20

**Authors:** Vianney Gilard

**Affiliations:** Department of Spine Surgery, Clinique Saint Hilaire, 76000 Rouen, France; vianney.gilard@normandie-rachis.fr

**Keywords:** neurosurgery, maximal safe resection, functional preservation, intraoperative mapping, neurocognitive outcomes, quality of life, patient-reported outcomes, connectomics, artificial intelligence, value-based care

## Abstract

Over the past decades, neurosurgery has undergone a profound transformation driven by technological innovation and a paradigm shift toward patient-centered outcomes. Historically evaluated through mortality rates and extent of resection, modern neurosurgery increasingly prioritizes preservation of neurological function, cognitive integrity, and quality of life. Innovations such as intraoperative mapping, multimodal neuromonitoring, image-guided surgery, minimally invasive techniques, and enhanced recovery protocols have reshaped surgical decision-making. However, the true value of these advancements lies in their measurable impact on clinically meaningful outcomes. This narrative review examines how technical progress translates into functional, cognitive, and quality-of-life benefits, while critically discussing current limitations of evidence and future directions toward personalized, outcome-driven neurosurgery.

## 1. Introduction—A Paradigm Shift in Modern Neurosurgery

Neurosurgery has traditionally been assessed through survival metrics, complication rates, and extent of lesion removal [1]. While these parameters remain essential, they no longer capture the full spectrum of surgical success. Advances in neuroimaging, intraoperative monitoring, and perioperative care have enabled a transition from “maximal resection” to “maximal safe resection” [1,2], emphasizing functional preservation [3].

Today, surgical excellence is increasingly defined by postoperative independence, cognitive outcomes, and patient-reported quality of life [4,5,6]. This shift reflects broader changes in medicine toward value-based and patient-centered care [7,8]. Although diffuse glioma represents one of the most extensively studied paradigms in which the balance between extent of resection and functional preservation has been quantitatively evaluated, the conceptual shift toward patient-centered outcome assessment extends across multiple neurosurgical domains. Similar considerations apply to skull base tumors, vestibular schwannoma, brain metastases, and other intracranial lesions in which treatment decisions must balance oncological benefit with preservation of neurological function, cognition, and quality of life. Glioma surgery is therefore discussed as an illustrative model through which the principles of maximal safe resection and outcome-driven decision-making have been most extensively characterized.

This narrative conceptual review aims to connect concrete neurosurgical innovations with clinically meaningful outcomes, highlighting both achievements and remaining challenges. Rather than providing a systematic synthesis of all the available literature, this review focuses on key conceptual advances that illustrate the evolving relationship between technological progress and patient-centered outcomes. The literature discussed in this review was identified through targeted searches in PubMed/MEDLINE and Web of Science, with emphasis on influential prospective studies, meta-analyses, and landmark contributions addressing functional preservation, neurocognitive outcomes, and quality of life. Priority was given to studies published over the past two decades that have shaped contemporary understanding of outcome-driven neurosurgery. Reference selection was guided by conceptual relevance rather than predefined systematic inclusion criteria.

## 2. Evolution of Neurosurgical Techniques: An Outcome-Oriented Perspective

Modern neurosurgery evolved from an era of anatomical exploration to precision-guided intervention. Early microsurgical techniques introduced by pioneers such as Gazi Yasargil revolutionized operative visualization [9]. Subsequent integration of neuronavigation and functional imaging further refined surgical planning [10,11]. The conceptual transition from aggressive tumor removal to maximal safe resection has been particularly evident in glioma surgery. Studies have demonstrated that survival benefit must be balanced against functional preservation, especially in eloquent brain regions [1,2,12]. In parallel, outcome assessment has expanded to include neurocognitive function, long-term autonomy, and social reintegration. This broader perspective has redefined surgical success [13,14,15]. Awake craniotomy with cortical and subcortical mapping has become a cornerstone in surgery for tumors involving eloquent areas. Direct electrical stimulation enables real-time identification of motor, sensory, and language pathways, significantly reducing permanent deficits [14,16].

Multimodal neuromonitoring—including motor evoked potentials (MEP), somatosensory evoked potentials (SEP), and continuous language testing—provides dynamic feedback during resection [17,18,19]. Evidence suggests that these techniques decrease postoperative motor deficits and improve long-term independence. Importantly, functional recovery is not solely dependent on preservation but also on neuroplasticity [1,15]. Awake mapping strategies facilitate individualized resection boundaries tailored to each patient’s functional architecture [20].

It should be acknowledged that a substantial proportion of evidence supporting awake mapping and multimodal neuromonitoring derives from high-volume academic centers with specialized expertise. Outcomes may therefore vary across institutions depending on surgical experience, multidisciplinary infrastructure, and case selection. This variability highlights the importance of structured training and careful implementation when translating advanced functional techniques into broader clinical practice. Long-term follow-up studies suggest that functional mapping strategies contribute to sustained preservation of language and motor function, facilitating higher rates of return to work and long-term functional independence in selected patient populations.

## 3. Image-Guided and Intraoperative Imaging Techniques

Neuronavigation systems have progressively transformed neurosurgical planning by integrating high-resolution preoperative MRI with functional imaging modalities such as functional MRI (fMRI) and diffusion tensor imaging (DTI) tractography. These technologies enable three-dimensional anatomical reconstruction and visualization of eloquent white matter tracts, improving surgical orientation and reducing the risk of injury to critical cortical and subcortical structures [21,22]. DTI-based fiber tracking, in particular, has enhanced the identification of corticospinal tracts and language pathways, facilitating safer resections in tumors located near eloquent regions [16,22].

Beyond preoperative guidance, intraoperative imaging modalities—including intraoperative MRI (iMRI) and intraoperative ultrasound (iUS)—provide dynamic real-time assessment of residual tumor tissue. Randomized evidence has demonstrated that iMRI significantly increases the extent of resection in glioma surgery compared with conventional neuronavigation alone [23]. Subsequent observational and cohort studies have suggested that greater extent of resection achieved with intraoperative imaging may translate into improved progression-free survival, although its impact on overall survival remains less consistently demonstrated [12]. Intraoperative ultrasound has emerged as a cost-effective and flexible alternative, offering real-time feedback and compensating for brain shift during resection [24].

However, imaging-guided precision must be interpreted within functional constraints. Maximizing radiological extent of resection does not uniformly equate to improved patient-centered outcomes. Excessively aggressive resection in eloquent areas may result in permanent neurological deficits that negate oncological benefit and negatively impact quality of life [13,15]. Consequently, modern surgical strategy increasingly combines advanced imaging with functional mapping techniques to balance oncological radicality and neurological preservation within a patient-specific framework. Although increased extent of resection has been consistently demonstrated with intraoperative imaging technologies, evidence regarding their impact on long-term survival and quality of life remains heterogeneous, highlighting the need to interpret radiological outcomes within a broader functional framework.

## 4. Minimally Invasive Approaches and Surgical Morbidity

Minimally invasive neurosurgery—including endoscopic, tubular, and keyhole approaches—aims to reduce soft tissue disruption, limit brain retraction, and accelerate postoperative recovery while maintaining oncological efficacy. These techniques leverage improved optical systems, angled endoscopes, and refined instrumentation to enhance visualization through smaller surgical corridors [25,26]. In selected patients, this reduction in approach-related morbidity translates into decreased postoperative edema, shorter intensive care stay, and faster return to functional independence.

Endoscopic endonasal surgery (EES) has fundamentally transformed skull base tumor management, particularly for pituitary adenomas and selected anterior skull base lesions. Compared with traditional transcranial approaches, EES has been associated with reduced brain manipulation, lower rates of cosmetic morbidity, and improved visual outcomes in appropriately selected cases. Multicenter series have also demonstrated comparable or improved extent of resection with acceptable complication profiles, although cerebrospinal fluid (CSF) leak remains a specific risk requiring technical expertise [27,28].

Similarly, minimally invasive tubular retractors and neuroendoscopic-assisted techniques minimize cortical and subcortical injury by distributing retraction forces and preserving surrounding white matter tracts. Comparative studies suggest shorter hospital stays, reduced postoperative pain, and earlier mobilization in carefully selected patients undergoing minimally invasive approaches for intracranial and spinal lesions [29,30]. However, these benefits depend strongly on surgeon experience and institutional volume.

While minimally invasive techniques aim to reduce approach-related morbidity, surgical outcomes are not determined solely by intraoperative strategy. Perioperative management plays a critical complementary role in shaping recovery trajectories and long-term functional outcomes. This broader perspective has contributed to the emergence of structured perioperative optimization pathways such as Enhanced Recovery After Surgery (ERAS) protocols.

## 5. Enhanced Recovery and Perioperative Optimization in Neurosurgery

Enhanced Recovery After Surgery (ERAS) protocols have been progressively adopted in neurosurgery as part of a broader shift toward value-based perioperative care [31]. Originally developed in colorectal surgery, ERAS pathways integrate multimodal analgesia, opioid-sparing strategies, early mobilization, optimized fluid therapy, prevention of postoperative nausea and vomiting, and standardized discharge criteria. In cranial and spinal procedures, implementation of structured ERAS programs has been associated with reduced postoperative complications, shorter length of stay (LOS), and lower readmission rates without increasing adverse events [32,33,34].

In elective cranial surgery, ERAS protocols emphasizing early ambulation, early oral intake, and avoidance of prolonged intensive care monitoring have demonstrated meaningful reductions in LOS—often by 1–2 days—while maintaining safety profiles comparable to conventional pathways [34,35]. Similarly, in spine surgery, ERAS-based approaches combining minimally invasive techniques, multimodal analgesia, and structured rehabilitation have been linked to reduced opioid consumption, earlier mobilization, and improved patient-reported recovery trajectories [32,36]. These improvements translate into higher patient satisfaction and faster return to baseline functional status in appropriately selected populations.

Importantly, ERAS implementation extends beyond individual perioperative measures and reflects a systems-based reorganization of care. Standardized pathways reduce variability in clinical practice, facilitate multidisciplinary coordination, and enable more predictable recovery patterns. From a health-economic perspective, reduced LOS and complication rates may improve cost-effectiveness, particularly in high-volume centers [37].

However, challenges remain. Evidence in neuro-oncology is less robust than in spine surgery, and heterogeneity in protocol components limits cross-study comparisons. The strength of evidence supporting ERAS protocols differs between neurosurgical subspecialties. In spine surgery, multiple prospective studies and systematic reviews have demonstrated consistent reductions in length of stay, opioid consumption, and complication rates. In cranial neurosurgery, available data are increasing but remain comparatively heterogeneous, with fewer randomized studies and greater variability in protocol composition. These differences highlight the need for further standardization and prospective evaluation of ERAS pathways in neuro-oncology.

Furthermore, successful ERAS adoption requires institutional commitment, team training, and continuous auditing. Despite these limitations, perioperative optimization represents a critical complement to intraoperative innovation, reinforcing the concept that surgical success is determined not only by technical precision but also by structured recovery and long-term functional outcomes.

## 6. Measuring and Defining Meaningful Outcomes in Modern Neurosurgery

The evaluation of neurosurgical success has historically relied on metrics such as extent of resection, postoperative complications, and overall survival. While these indicators remain indispensable, they provide an incomplete representation of what ultimately matters to patients. Radiological completeness of resection does not necessarily equate to preserved independence, cognitive integrity, or long-term quality of life. Interpretation of surgical outcomes must consider the interaction between resection strategy and adjuvant therapies such as radiotherapy and chemotherapy. Postoperative neurological deficits may limit eligibility for adjuvant treatment, thereby indirectly influencing survival outcomes. Conversely, anticipated responsiveness to systemic therapy may influence surgical risk tolerance. Modern neuro-oncology increasingly relies on integrated treatment strategies in which surgical planning is coordinated with oncological management to optimize both survival and functional outcomes.

As survival improves—particularly in low-grade glioma and selected high-grade glioma subgroups—the relevance of functional and patient-centered outcomes becomes increasingly central [1,12].

### 6.1. Beyond Extent of Resection: The Limits of Traditional Metrics

Extent of resection (EOR) has long been considered a surrogate marker for oncological success. Meta-analyses have demonstrated an association between greater EOR and improved survival in glioblastoma and lower-grade gliomas [15]. However, this relationship is not linear and may be confounded by tumor biology, location, and patient selection. Importantly, aggressive resection in eloquent regions can result in neurological deficits that significantly diminish quality of life, functional independence, and even long-term survival due to reduced eligibility for adjuvant therapies [3].

Binary definitions of postoperative deficits (deficit vs. no deficit) further oversimplify complex neurological outcomes [38,39]. Subtle impairments in executive function, language fluency, attention, or processing speed may not be captured by routine neurological examination yet can profoundly affect social reintegration and professional activity [4,40]. Therefore, a paradigm shift is required: surgical precision must be evaluated not only by how much tumor is removed, but by how well the patient functions afterward.

### 6.2. Neurocognitive Outcomes and the Concept of “Invisible Deficits”

Neurocognitive impairment is increasingly recognized as a critical endpoint in neuro-oncology. Long-term studies in low-grade glioma patients have demonstrated that treatment-related cognitive changes—whether from surgery, radiotherapy, or chemotherapy—may emerge months or years after intervention [41]. Executive dysfunction, working memory impairment, and reduced processing speed can compromise employability and social autonomy even in patients considered neurologically intact.

Functional MRI and advanced neuropsychological testing have revealed that subtle network-level disturbances may persist despite preserved motor and language function. These “invisible deficits” challenge the traditional surgical focus on overt neurological preservation. The concept of connectome-based surgery emphasizes that cognitive function is supported by distributed networks rather than isolated cortical regions [13,42]. Consequently, outcome assessments must integrate standardized neuropsychological batteries capable of detecting domain-specific changes over time.

Return-to-work rates and long-term autonomy are particularly relevant functional markers. Studies have shown that even minor postoperative deficits can significantly reduce return-to-work probability, underscoring the socioeconomic impact of neurosurgical decision-making [43]. Incorporating these endpoints into clinical evaluation aligns surgical goals with real-world patient priorities.

### 6.3. Patient-Reported Outcomes and Quality of Life

Patient-Reported Outcome Measures (PROMs) have emerged as essential tools for capturing dimensions of health not visible to clinicians [44,45]. Instruments such as the EORTC QLQ-C30 and its brain tumor-specific module (BN20) allow standardized assessment of fatigue, emotional functioning, cognitive complaints, and social participation [5,46]. The Response Assessment in Neuro-Oncology Patient-Reported Outcome (RANO-PRO) initiative further underscores the necessity of integrating PROMs into neuro-oncological trials [5].

Despite increasing adoption, PROM utilization remains heterogeneous across studies. Differences in timing, instrument selection, and reporting standards limit cross-trial comparability. Furthermore, many studies assess outcomes only in the early postoperative period, failing to capture delayed cognitive decline or adaptive recovery processes [47,48].

The integration of PROMs into routine neurosurgical practice remains uneven. However, their incorporation is essential for a patient-centered evaluation framework. In this context, surgical success must be defined not only by tumor control but by sustained quality of life.

Despite their recognized value, implementation of routine neuropsychological assessment and PROM collection presents practical challenges in daily clinical practice. Time constraints, limited access to trained neuropsychologists, variability in assessment tools, and administrative burden may limit systematic data collection. Digital assessment platforms and shorter validated instruments may facilitate broader integration of patient-reported outcomes into routine care, but further efforts are required to standardize workflows and ensure feasibility across diverse healthcare settings.

### 6.4. Methodological Challenges and the Need for Standardization

A major barrier to meaningful outcome assessment lies in methodological heterogeneity. Cognitive outcomes are measured using variable neuropsychological batteries [49]; motor deficits are often dichotomized; and timing of assessment ranges from immediate postoperative to years later. This variability impedes meta-analysis and prevents establishment of universal benchmarks.

Moreover, randomized controlled trials (RCTs) in surgical neuro-oncology are rare. Ethical considerations, surgeon expertise variability, and patient heterogeneity limit feasibility [50]. Consequently, much of the evidence supporting technological innovations derives from observational studies conducted in high-volume academic centers [51,52]. While informative, these designs are vulnerable to selection bias and center effects.

To advance toward outcome-driven neurosurgery, consensus definitions and standardized assessment protocols are urgently required. International collaborations such as RANO and ERAS Society initiatives provide promising frameworks, yet broader adoption is necessary [6,37]. Ultimately, technological innovation must be accompanied by methodological rigor to ensure that improvements in precision translate into measurable patient benefit. An additional methodological challenge relates to the timing of neurocognitive assessment. Reliable interpretation of cognitive outcomes requires standardized evaluation timepoints, including preoperative baseline testing and longitudinal follow-up assessments. Without baseline reference values, postoperative changes may be underestimated or misinterpreted, particularly in patients presenting with pre-existing tumor-related cognitive impairment. Standardized follow-up intervals are therefore essential to distinguish transient postoperative effects from persistent or delayed cognitive changes.

## 7. From Innovation to Value: Structural Barriers and the Future of Outcome-Driven Neurosurgery

Technological innovation in neurosurgery has progressed rapidly over the past decades. However, the translation of technical advancements into measurable patient-centered benefits remains uneven. Increased precision, improved visualization, and expanded intraoperative monitoring capabilities do not automatically translate into better long-term functional or quality-of-life outcomes [7,53]. Bridging this gap requires addressing structural, methodological, and systemic challenges that continue to shape contemporary neurosurgical practice.

### 7.1. Methodological and Structural Barriers

Randomized controlled trials (RCTs) in surgical neuro-oncology remain uncommon. Ethical constraints, tumor heterogeneity, surgeon-dependent expertise, and rapid technological evolution complicate traditional trial design [50,54]. Although intraoperative MRI has been shown to increase extent of resection in randomized settings, direct evidence linking this improvement to overall survival or sustained quality-of-life benefit is less robust [23]. Similarly, awake mapping strategies are widely accepted for eloquent gliomas, yet much of the supporting evidence derives from prospective cohorts or matched retrospective studies rather than randomized comparisons.

Another critical issue is the “center effect.” Advanced technologies are frequently evaluated in high-volume academic institutions with specialized expertise. Volume–outcome relationships have been demonstrated in neurosurgical oncology, suggesting that outcomes achieved in expert centers may not be generalizable to lower-volume settings [51,55]. This raises important questions regarding external validity and equitable access to innovation.

Economic considerations further complicate implementation. Technologies such as intraoperative MRI, advanced neuronavigation platforms, and emerging AI-based tools entail substantial financial investment. Cost-effectiveness analyses remain limited, and resource allocation increasingly demands demonstration of value—not merely technical feasibility [56]. A value-based neurosurgical model requires integrating survival, functional outcomes, and economic sustainability into a unified evaluation framework [57].

### 7.2. Toward Predictive and Network-Based Surgery

Future neurosurgery is likely to be shaped by a transition from reactive decision-making to predictive modeling. Connectome-based approaches conceptualize the brain as a dynamic network rather than a collection of isolated eloquent areas. Diffusion tractography and functional connectivity analyses allow individualized risk stratification, particularly in tumors involving frontal and language networks [58]. By anticipating potential network disruption, surgeons may better balance oncological ambition with cognitive preservation.

Artificial intelligence and machine learning models are increasingly applied to neuro-oncological datasets. By integrating imaging features, molecular markers, and clinical variables, predictive algorithms may estimate postoperative deficit risk, likelihood of recovery, recurrence probability, and survival trajectories. Preliminary studies suggest improved predictive performance compared with conventional statistical approaches, although external validation and prospective integration into routine workflows remain limited [59,60].

Molecular stratification further refines surgical strategy. The 2021 WHO classification of CNS tumors [61] has reinforced the central role of molecular markers in guiding treatment strategies. IDH mutation status, 1p/19q codeletion, TERT promoter mutation, CDKN2A/2B deletion, EGFR amplification, ATRX alteration, and histone mutations such as H3 K27M and H3 G34 define biologically distinct tumor entities with different growth patterns and prognostic trajectories. These molecular features increasingly influence the balance between oncological benefit and functional risk when determining the optimal extent of resection. For example, IDH-mutant gliomas often demonstrate prolonged survival, increasing the relative importance of long-term cognitive preservation, whereas more aggressive molecular profiles may justify different risk–benefit considerations. The integration of molecular and imaging features represents an important step toward personalized surgical strategy.

Despite promising early results, most predictive models remain derived from retrospective datasets and require robust external validation across diverse clinical settings. Prospective evaluation and integration into clinical workflows are necessary before widespread adoption can be recommended, ensuring that algorithmic predictions translate into meaningful improvements in patient outcomes.

Several limitations must be considered before widespread implementation of AI-based predictive models in neurosurgery. Many algorithms are developed using retrospective datasets that may not fully capture real-world heterogeneity, raising concerns regarding external validity and reproducibility. Model performance may also be influenced by dataset imbalance, imaging variability, and differences in institutional practice patterns, potentially introducing bias. Furthermore, integration of predictive tools into clinical workflows requires user-friendly interfaces, interpretability, and prospective validation demonstrating added clinical value beyond conventional decision-making frameworks.

### 7.3. Longitudinal Monitoring and Integrated Care Models

Outcome-driven neurosurgery extends beyond intraoperative precision. Wearable devices, digital cognitive platforms, and remote monitoring technologies offer opportunities for longitudinal assessment of fatigue, cognitive performance, and functional activity. Continuous outcome tracking may allow earlier detection of decline and more timely intervention, moving evaluation beyond episodic clinic visits [62,63].

Equally important is the integration of multidisciplinary care. Surgery should be conceptualized as one component within a broader functional recovery continuum that includes neuropsychological support, rehabilitation, oncological management, and structured return-to-work programs [64]. Early cognitive rehabilitation and individualized recovery planning may mitigate subtle deficits that are not apparent in routine neurological examinations.

As predictive tools and risk models evolve, ethical considerations become increasingly relevant. Determining acceptable risk thresholds, communicating probabilistic outcomes, and incorporating patient preferences into complex surgical decisions demand transparent shared decision-making frameworks [65]. Ultimately, the future of neurosurgery will not be defined solely by technological sophistication but by the capacity to align innovation with individualized, meaningful patient benefit. Outcome-driven neurosurgery should be conceptualized within an integrated care continuum that includes surgery, radiotherapy, systemic therapies, rehabilitation, and longitudinal neurocognitive monitoring. Multidisciplinary decision-making enables individualized therapeutic strategies that balance oncological control with preservation of neurological and cognitive function.

Although numerous technological innovations have reshaped modern neurosurgery, their level of evidence and measurable impact on functional and quality-of-life outcomes remain heterogeneous. Table 1 provides a comparative overview of major neurosurgical advancements, summarizing their evidence level, demonstrated outcome impact, and principal limitations. This synthesis underscores the need to align technological progress with standardized, patient-centered outcome evaluation.

## 8. Conclusions

Neurosurgery has evolved from an era defined by anatomical mastery and maximal tumor removal to one increasingly guided by functional preservation and patient-centered outcomes. Technological advances—ranging from intraoperative imaging and functional mapping to minimally invasive techniques and enhanced recovery pathways—have undeniably improved surgical precision and safety. Yet precision alone does not define success.

The true measure of modern neurosurgery lies in its ability to preserve cognitive integrity, sustain quality of life, and enable long-term autonomy. Radiological extent of resection must be interpreted within functional and psychosocial contexts. Innovation must be accompanied by rigorous outcome measurement, standardized reporting, and equitable implementation.

The next phase of progress will require integration rather than accumulation—combining network neuroscience, predictive analytics, molecular stratification, and longitudinal outcome monitoring within multidisciplinary care models. In this framework, technology becomes a means rather than an end: a tool serving the broader objective of value-based, outcome-driven neurosurgical care. A more comprehensive evaluation of findings from studies involving tumors beyond gliomas is warranted to determine the extent to which the present conclusions can be generalized.

Ultimately, the future of neurosurgery will be defined not by how much tumor is removed, but by how well patients live after surgery.

## Figures and Tables

**Table 1:** Major Neurosurgical Innovations and Outcome Impact.

Technology	Current Evidence Base (2025)	Demonstrated Outcome Impact	Key Limitations
Awake cortical/subcortical mapping	Prospective cohorts, meta-analyses; limited RCTs	↓ permanent language/motor deficits; improved return to work	Expertise bias; patient selection; limited RCT data
Multimodal neuromonitoring (MEP/SEP/language)	Observational studies; systematic reviews	↓ postoperative motor deficits	Variable alarm criteria; heterogeneity
Intraoperative MRI	RCTs (extent of resection); cohort survival data	↑ extent of resection; possible ↑ PFS	High cost; unclear OS benefit
Intraoperative ultrasound	Cohort studies; growing prospective data	real-time tumor visualization; cost-effective	Operator-dependent
Minimally invasive/keyhole approaches	Comparative cohort studies	↓ LOS; ↓ postoperative pain	Not suitable for all lesions
Endoscopic endonasal surgery	Large multicenter cohorts	↓ morbidity in skull base tumors	Learning curve; CSF leak risk
ERAS protocols	Prospective studies; meta-analyses (2022–2024)	↓ LOS; ↓ complications; ↑ patient satisfaction	Implementation variability
AI-based predictive models	Retrospective ML validation studies (2023–2025)	improved risk stratification; personalized planning	External validation lacking
Connectome-guided surgery	Prospective observational studies	better cognitive preservation hypothesis	Early-stage evidence

## Data Availability

No new data were created or analyzed in this study. Data sharing is not applicable to this article.
